# Effects of β-hydroxy-β-methylbutyrate (HMB) supplementation on lipid profile in adults: a GRADE-assessed systematic review and meta-analysis of randomized controlled trials

**DOI:** 10.3389/fnut.2024.1451282

**Published:** 2024-09-25

**Authors:** Behrad Sadeghi, Hossein Bahari, Hannane Jozi, Mohammad-Ali Hasanzadeh, Dorna Hashemi, Mohammad Vesal Bideshki

**Affiliations:** ^1^Department of Agricultural, Forest and Food Science (DISAFA), University of Torino, Grugliasco, Torino, Italy; ^2^Transplant Research Center, Clinical Research Institute, Mashhad University of Medical Sciences, Mashhad, Iran; ^3^Student Research Committee, Tabriz University of Medical Sciences, Tabriz, Iran; ^4^Department of Immunology, School of Medicine, Jiroft University of Medical Sciences, Jiroft, Iran; ^5^Department of Food Science and Technology, Sarvestan Branch, Islamic Azad University, Sarvestan, Iran; ^6^Department of Biochemistry and Diet Therapy, School of Nutrition and Food Science, Tabriz University of Medical Sciences, Tabriz, Iran

**Keywords:** β-hydroxy-β-methylbutyrate, HMB, lipid profile, dyslipidemia, cardiovascular health, meta-analysis

## Abstract

**Background and aim:**

The regulation of lipid metabolism is crucial for preventing cardiovascular diseases, which are among the leading causes of mortality worldwide. β-hydroxy-β-methylbutyrate (HMB) has garnered attention for its potential role in modulating lipid profiles. However, the magnitude of these effects are unclear due to the heterogeneity of the studies. This study aimed to provide a comprehensive overview of the randomized controlled trials (RCTs) that have examined the effects of HMB on lipid profiles in adults.

**Methods:**

Databases including PubMed, Web of Science, and Scopus, were searched for relevant studies through January 2024. The study protocol was also registered at Prospero (no. CRD42024528549). Based on a random-effects model, we calculated WMDs and 95% confidence intervals (CIs). The outcomes assessed included total cholesterol (TC), triglyceride (TG), low-density lipoprotein cholesterol (LDL-C), and high-density lipoprotein cholesterol (HDL-C). Sensitivity, subgroup and meta-regression analyses were also conducted.

**Results:**

Our analysis included a total of 10 RCTs comprising 421 participants. The pooled data revealed no significant effect of HMB supplementation on TC (WMD: −2.26 mg/dL; 95%CI: −6.11 to 1.58; *p* = 0.25), TG (WMD: −2.83 mg/dL 95% CI: −12.93 to 7.27; *p* = 0.58), LDL-C (WMD: 0.13 mg/dL; 95%CI: −3.02 to 3.28; mg; *p* = 0.94), and HDL-C (WMD: −0.78 mg/dL; 95%CI: −2.04 to 0.48; *p* = 0.22). The quality of evidence was rated as moderate to low for all outcomes.

**Conclusion:**

The current evidence from RCTs suggests that HMB supplementation does not significantly alter lipid profiles, including TC, TG, LDL-C, and HDL-C. Further research is warranted to confirm these results and explore the potential mechanisms of action of HMB.

**Systematic review registration:**

https://www.crd.york.ac.uk/prospero/display_record.php?RecordID=528549, CRD42024528549.

## 1 Introduction

A metabolite of branched-chain amino acid (BCAA), β-hydroxy-β-methyl butyrate (HMB) is one of the sports supplements widely utilized by athletes for its anticatabolic effects during stressful situations (1). HMB is known to impact various aspects of muscle strength, muscle mass, muscle damage, and exercise recovery (2). Additionally, HMB has a key role in muscle quality by reducing the breakdown of muscle proteins (up to 57%), enhancing muscle protein synthesis (up to 70%), and increasing the stability of muscle membranes (3, 4). Furthermore, this supplement is associated with fat loss and body composition, which has made it one of the most popular supplements (5). As a result of these effects, HMB supplementation could potentially influence the presence and progression of metabolic syndrome, diabetes mellitus (DM), and cardiovascular disease (CVD), suggesting its significance in the broader context of metabolic health and chronic disease prevention (6, 7). In the process of cell growth and membrane repair, 3-hydroxy-3-methylglutarylcoA (HMG-CoA) is metabolized by HMB as the rate-limiting component of cholesterol synthesis (8). Consequently, HMB may contribute to cholesterol synthesis and lipid profiles (9). Assessing the lipid profile and identifying dyslipidemia characterized by lower levels of high-density lipoprotein (HDL), higher levels of low-density lipoprotein (LDL), total cholesterol (TC), and total triglyceride (TG) of serum is very necessary (10). Maintaining optimal blood lipid levels can help prevent chronic kidney disease (CKD), CVD, and DM (10, 11).

Several studies have demonstrated that supplementation with HMB may decrease LDL levels and subcutaneous fat deposition by reducing cholesterol levels (12, 13). It was shown by Nisen et al. that HMB supplementation significantly reduced serum TC (5/8%) and LDL (7/3%). The main point is that decreasing LDL levels among individuals with high cholesterol levels by HMB supplementation is more effective than the other methods (14). In addition, fat utilization, fatty acid oxidation, and the generation of fatty acids in human skeletal muscle cells and adipocytes are facilitated by HMB supplementation, leading to a decrease in TG levels of serum (15, 16). Nevertheless, despite prior studies, a number of studies dispute the efficacy of HMB in improving lipid profiles (17–19). Moreover, further research is needed to explore new methods and supplements designed to influence blood lipid levels (20, 21).

Overall, not only given the limited and conflicting data regarding the impact of HMB on lipid profiles, but also meta-analysis and systematic review studies about this subject are not available. Hence, this study was conducted to investigate the potential effects of HMB supplementation on the lipid profile of adults.

## 2 Methods

This meta-analysis was conducted in accordance with the PRISMA (Preferred Reporting Items for Systematic Reviews and Meta-Analyses statement) guidelines (22) and was registered with the International Prospective Register of Systematic Reviews (PROSPERO, CRD42024528549).

### 2.1 Search strategy

A comprehensive literature search of the PubMed, Scopus, and Web of Science online databases was conducted until January 12, 2024, to identify articles evaluating the effect of HMB supplements on blood lipid parameters. To ensure coverage of the latest publications, email alerts were set to notify us of any new articles listed in these databases. The detailed search strategy is presented in Supplementary Table S1. Two researchers (B. S. and H. J.) separately hand-searched the reference lists of the related review papers, meta-analyses, and randomized clinical trials (RCTs) along with Google Scholar (using search phrases) to find additional RCTs.

### 2.2 Study selection

Two authors (B.S and H.J) separately screened the titles and abstracts of all the identified papers in order to determine their eligibility based on our inclusion criteria. Discrepancies were resolved by discussion with MV.B. To ensure a comprehensive and reproducible search strategy, all identified articles were managed using EndNote, a reference management software. This tool was instrumental in organizing the identified articles, removing duplicates, and facilitating the screening process. Furturemore, attempts were made to locate the full text of all related articles through open access databases and university library subscriptions. When these resources were unavailable, the corresponding authors were contacted directly to request a copy for analysis. The following criteria for inclusion were used to select studies for analysis based on the Population, Intervention, Comparison, Outcome (PICO) presented in Table 1: (1) RCTs with a crossover or parallel design; (2) studies involving adults (aged ≥18 years); (3) intervention durations with HMB supplements of at least 2 weeks; (4) included suitable intervention and control groups; (5) had sufficient information on lipid indices with mean or median values (standard errors (SEs), standard deviations (SDs), or 95% confidence intervals (Cls)) for the intervention and control groups at baseline and at the end of the intervention; and (6) full text of the article available in the English language.

**Table 1:** PICOS criteria for inclusion and exclusion of studies.

Parameter	Criteria
Participant	Adults
Intervention	β-Hydroxy-β-Methylbutyrate
Comparator	Matched control group
Outcomes	Total cholesterol, Triglycerides, Low-density lipoprotein cholesterol, High-density lipoprotein cholesterol
Study design	Controlled clinical trial

We excluded studies based on the following criteria: (1) it was not possible to calculate a net benefit of HMB supplementation; (2) the duration of supplementation was less than 2 weeks; (3) the participants were not adults (18 > years of age); (4) studies with animal, case–control, cross-sectional, cohort designs, or review papers; and (5) the data on the baseline and follow-up lipid profile values were inadequate.

### 2.3 Data extraction

Two authors (B.S and H.J) separately selected eligible articles based on our inclusion and exclusion criteria. The extracted data were double-checked by (MV.B, H.J, and B.S) and agreed upon by all authors.

An extract of the data was made, including: author names, study location, year of publication, participant characteristics (sex, health status, age, and baseline BMI), intervention and control group sample sizes, dosage and type of supplementation, and co-interventions. A comparison of the baseline and final data for blood lipid indices was made. To ensure consistency, lipid index measurements originally reported in units other than mg/dL were converted and subsequently included in the analysis. In cases where results were reported at multiple measurements, only the measurements at baseline and at the end of the intervention duration were considered. If a study used different doses of HMB, each dose was analyzed separately.

### 2.4 Assessment of quality and certainty

The risk of bias in the included studies was assessed using the Cochrane Risk of Bias 2 (ROB2) tool (23). The following assessment domains were considered when assessing bias in the included studies: (1) adequacy of sequence generation (selection bias), (2) allocation sequence concealment (selection bias), (3) blinding (performance bias), (4) blinding of outcome assessment (detection bias), (5) addressing of dropouts (attrition bias), (6) reporting selective outcomes (reporting bias), and (7) other potential sources of bias. Two authors (B. S. and H. J.) assessed the studies and assigned “Low,” “High,” or “Unclear” for each domain. In the event of any disagreements, a third author (MV. B) was consulted to reach an agreement. The GRADE (Grading of Recommendations Assessment, Development, and Evaluation) protocol (24) developed by the GRADE Working Group^1^ was utilized to assess the certainty of evidence. GRADE introduces a systematic and consistent framework for assessing the quality and validity of evidence derived from trials (25). This assessment resulted in the categorization of certainty into four levels: High, Moderate, Low, or Very Low. The GRADE approach evaluates the certainty of evidence based on five key criteria: (1) risk of bias, (2) inconsistency, (3) indirectness, (4) imprecision, and (5) publication bias.

### 2.5 Statistical analysis

We analyzed the effects of HMB supplementation on the following outcomes: (1) total cholesterol (mg/dL), (2) HDL-C (mg/dL), (3) LDL-C (mg/dL), and (4) triglycerides (mg/dL). Weighted mean differences (WMDs) and 95% Cls were used to express effect sizes. The mean and SD values of these blood lipid indices at baseline and at the end of the intervention were extracted from the papers. If the mean difference was not reported, we used the following formulas to calculate it: (value at the endpoint of the intervention group - value at the baseline of the intervention group) and (value at the endpoint of the control group - value at the baseline of the control group). In the event that SD of mean difference was not reported, we used the following formula to calculate it: $SD=\mathrm{square}\;\mathrm{root}\;[{\left(SD\;\mathrm{pretreatment}\right)}^{2}+$ ${\left(SD\;\mathrm{posttreatment}\right)}^{2}–\left(2R\times SD\;\mathrm{pretreatment}\times SD\;\mathrm{posttreatment}\right)]\text{,}$ in which R (correlation coefficient) of 0.5 was assumed (26). In studies where SE was reported instead of SD, we used the following formula to calculate it: SD = SE × square root (*n*), where *n* is the number of subjects. Medians were converted into means, and studies that reported 95% CIs, interquartile ranges (IQRs), and SEs were converted to SDs, all using the methods developed by Hozo et al. (27). Cochran’s Q-test and the $I^{2}$ test were conducted to calculate between-study heterogeneity, with values of *p* < 0.1 and an $I^{2}$ value (≥ 50%) indicating significant heterogeneity. Based on heterogeneity, the results were presented either using the random effects model or using the fixed effects model. For the purpose of analyzing the effect size of each trial, we used a leave-one-out sensitivity analysis, in which one trial was excluded from each analysis. In addition, a subgroup analysis was conducted to evaluate the influence of factors such as different types of HMB supplements, dosage, supplementation duration, baseline blood lipid concentrations, BMI, and health status on study outcomes. Funnel plots, Egger’s weighted regression, and Begg’s rank correlation tests were utilized to identify potential publication bias (28). Statistical analysis was conducted using STATA for Windows version 16.0 (Stata Corporation, College Station, TX, United States). The level of significance was determined as *p* < 0.05.

## 3 Results

### 3.1 Study selection

**Figure 1:**
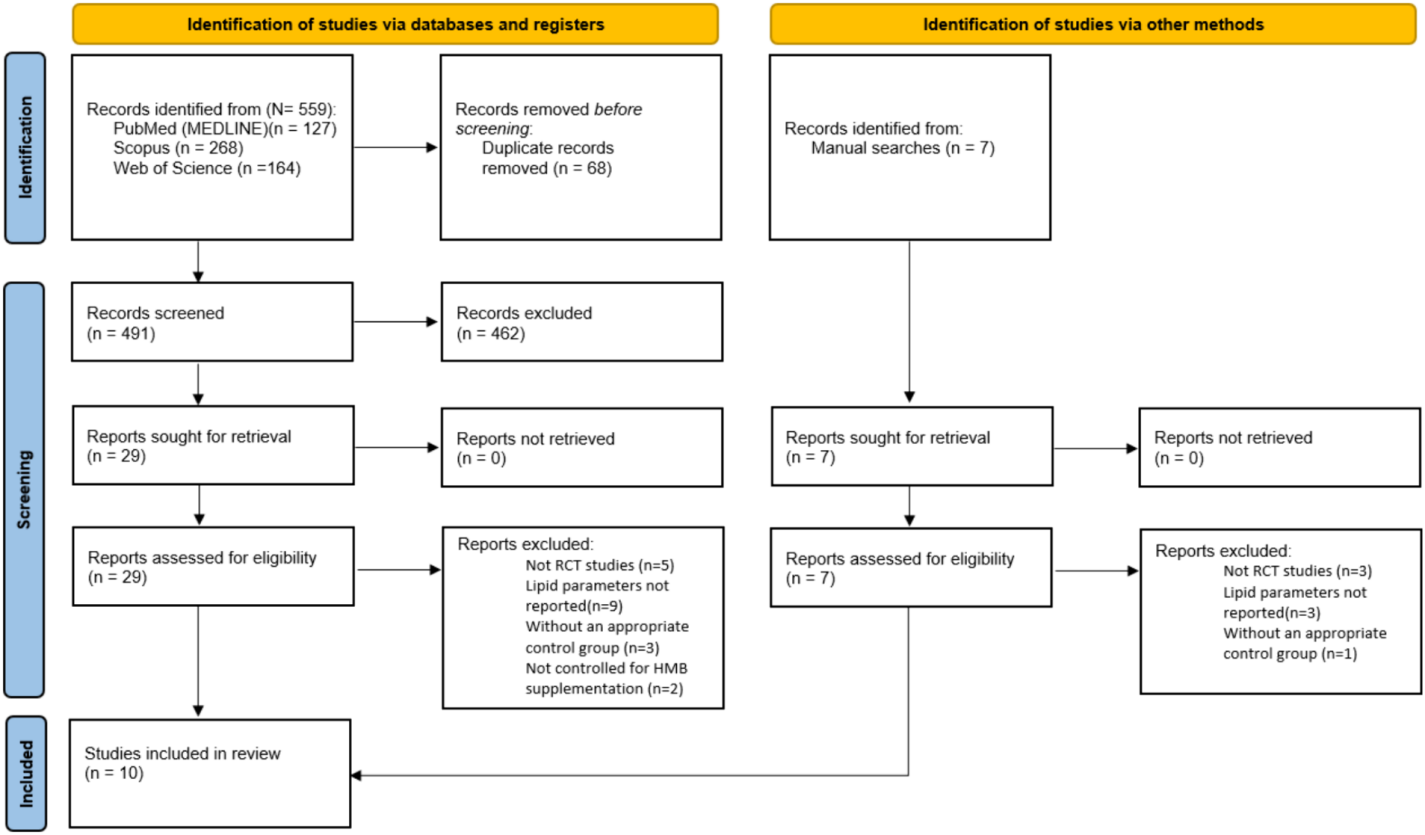
PRISMA 2020 flow diagram of databases searches, registers and other sources.

In Figure 1, it can be seen that the search protocol initially yielded a total of 559 studies. Out of these, 68 duplicates were identified and subsequently removed. Following this, an evaluation of the titles and abstracts based on the inclusion criteria resulted in the exclusion of 462 studies that were deemed irrelevant to the subject. A thorough assessment of the full texts of 29 studies led to the removal of 19 studies due to insufficient data reporting. Ultimately, 10 studies with 13 treatment arms were included in this meta-analysis.

### 3.2 Study characteristics

Table 2 provides information on the inclusion of 10 studies in this meta-analysis. All qualified articles included in this analysis were published between 2000 and 2023. The qualified studies were conducted in several countries, including Japan (29), China (30), Iran (31, 32), Brazil (33), Spain (34, 35), Australia (36), and the United States (37, 38). Five studies were conducted on men (31–33, 36, 38), one study was executed on women (29), and the others were conducted on both (30, 34, 35, 37). The intervention periods in the included trials ranged from 2 weeks (32) to 24 weeks (33). The supplement type was HMB in four studies (32–34, 38) and HMB-Ca in the other studies (29–31, 35–37). The dosage of HMB supplements in the included studies ranged from 1500 (29) to ~6000 (38) mg/day. The participants in these trials represented various populations, including healthy individuals (37, 38), patients with liver cirrhosis and clinical malnutrition (35), normally nourished non-cystic-fibrosis patients with bronchiectasis (34), Australian National Rugby League Team (36), elite canoeists volunteered (33), amateur athletes (31), beginner bodybuilders (32), older women with low muscle mass (29), and older adults (>60) with sarcopenia (30).

**Table 2:** Demographic characteristics of the included studies.

Studies	Country	Study design	Participant	Sex	Sample size		Trial duration (Week)	Means age		Means BMI		Intervention		
					IG	CG		IG	CG	IG	CG	Type	Dose (mg/day)	Control group
Yang et al. 2023	China	Ct, R, Db	old adults (>60) with sarcopenia	both	18	16	12 weeks	72.89	71.44	NR	NR	HMB-Ca + supervised resistance training	3,000	P + supervised resistance training
Espina et al. 2022	Spain	Ct, R, Db, Pa	patients with liver cirrhosis and clinical malnutrition	both	22	21	8 weeks	60.4	61.4	25.4 ± 4.66	26.3 ± 3.62	HMB-Ca + BCAA supplementation	3,000	P + BCAA supplementation
Osuka et al. 2021 (a)*	Japan	Ct, R, Db	older woman	woman	36	35	12 weeks	71.5	71.6	20.1 ± 2.2	20.7 ± 2.2	HMB-Ca + education	1,500	P + education
Osuka et al. 2021 (b)	Japan	Ct, R, Db	older woman	woman	36	37	12 weeks	73.5	71.8	21.3 ± 2.2	20.9 ± 2.1	HMB-Ca + exercise	1,500	P + exercise
Ansari et al. 2021	Iran	Ct, R	beginner bodybuilder	man	20	20	2 weeks	22.45	23.45	24.94 ± 1.96	25.21 ± 1.37	HMB	3,000	P
Olivera et al. 2016	Spain	Ct, R, Sb, Pa	normally nourished non-cystic-fibrosis patients with bronchiectasis	both	15	15	12 weeks	58.4	53.7	25.9 ± 12.9	27.3 ± 5.8	HMB + supervised exercise program and unsupervised session	1,500	supervised exercise program and unsupervised session
Arazi et al. 2015	Iran	Ct, R, Db	Amateur Athletes	man	10	10	4 weeks	22.4	22.7	24.1 ± 1.9	23.6 ± 1.5	HMB-Ca	3,000	P
Deutz et al. 2013*	USA	Ct, R, Db, Pa	healthy older adults confined to complete bed rest	both	11	8	8 weeks	67.4	67.1	24.9 ± 1.0	26.5 ± 1.2	HMB-Ca + resistance training	3,000	P
Ferreira et al. 2013	Brazil	Ct, R, Db	elite canoeists volunteered	man	14	6	24 weeks	NR	NR	NR	NR	HMB	37.5 mg/kg	P
Crowe et al. 2003 (a)	Australia	Ct	Australian National Rugby League team	man	11	6	6 weeks	24.9	24.9	NR	NR	HMB-Ca	3,000	NR
Crowe et al. 2003 (b)	Australia	Ct	Australian National Rugby League team	man	11	6	6 weeks	24.9	24.9	NR	NR	HMB-Ca + Cr	3,000	NR
Gallagher et al. 2000 (a)*	USA	Ct, R, Db	healthy untrained male	man	12	14	8 weeks	21	22.3	NR	NR	HMB + resistance training program	~3,000 (38 mg/kg/d)	resistance training program
Gallagher et al. 2000 (b)	USA	Ct, R, Db	healthy untrained male	man	11	14	8 weeks	21.8	22.3	NR	NR	HMB + resistance training program	~6,000 (76 mg/kg/d)	resistance training program

IG, intervention group; CG, control group; Pa, Parallel; Db, double-blinded; Ct, Controlled-trial; R, randomized; Placebo, P; NR, not reported; HMB, β-Hydroxy-β-Methylbutyrate; BCAA, branched-chain amino acid. *These studies report on separate populations. The participant numbers in each study were counted independently, with no overlap in the N values between these studies.

### 3.3 Quality assessment

In terms of the general risk of bias in the qualified articles, it was found that eight studies had a low risk of bias (29–33, 35, 37, 38), one study had a moderate risk of bias (34), and one article mentioned a high risk of bias (36). For transparency, traffic light graphs depicting the risk of bias for each outcome, as evaluated using the ROB2 tool, have been added to the Supplementary Figure S4. These graphs offer a clear visual representation of bias across the included studies, highlighting the different levels of risk for each domain.

### 3.4 Meta-analysis

#### 3.4.1 Effect of HMB supplementation on TC

**Figure 2:**
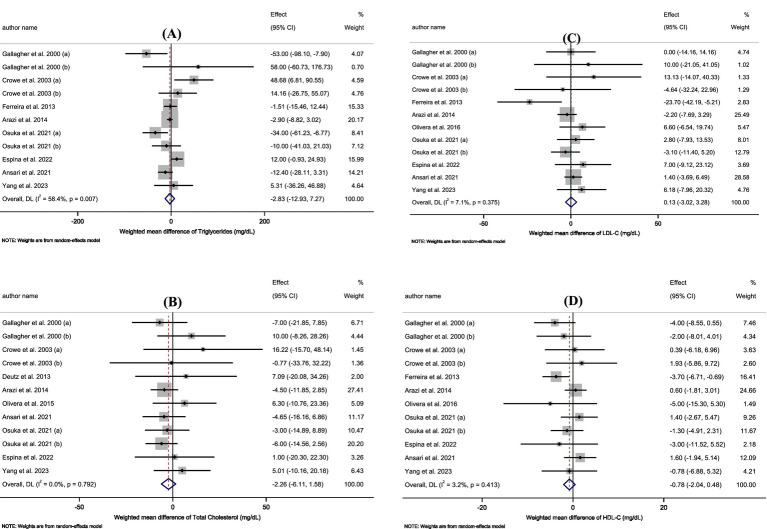
**(A–D)** Forest plot for the effect of HMB supplementation on **A**: Triglycerides, **B**: Total Cholesterol, **C**: LDL-C, **D**: HDL-C in adults, expressed as mean differences between intervention and control groups.

Pooled data from 12 effect sizes revealed no significant impact of HMB supplementation on TC levels compared to those in the control groups (WMD: −2.26 mg/dL; 95%CI: −6.11 to 1.58; *p* = 0.25; Figure 2A). Additionally, there was no significant heterogeneity among the included trials (I^2^ = 0.0%, *p* = 0.79). Subgroup analysis of HMB supplementation on TC is shown in Table 3.

**Table 3:** Subgroup analyses of HMB supplementation on lipid profile.

	Number of effect sizes	WMD (95%CI)	*p*-value	Heterogeneity		
				P heterogeneity	I^2^	P between sub-groups
HMB supplementation on Total cholesterol (mg/dL)						
Overall effect	12	−2.26 (−6.11, 1.58)	0.249	0.792	0.0%	
Baseline TC (mg/dL)						
<200	8	−1.97 (−6.90, 2.95)	0.433	0.662	0.0%	0.595
>200	3	−4.23 (−10.96, 2.49)	0.217	0.647	0.0%	
Trial duration (week)						
≤8	7	−2.58 (−7.79, 2.62)	0.33	0.625	0.0%	0.856
>8	5	−1.87 (−7.58, 3.83)	0.52	0.615	0.0%	
Intervention type						
HMB	6	−0.00 (−6.99, 6.99)	1.00	0.528	0.0%	0.448
HMB-Ca	6	−3.24 (−7.84, 1.36)	0.168	0.798	0.0%	
Gender						
Both	4	4.87 (−4.52, 14.26)	0.309	0.981	0.0%	0.238
Male	6	−2.95 (−8.26, 2.35)	0.275	0.567	0.0%	
Female	2	−4.97 (−11.92, 1.97)	0.16	0.688	0.0%	
Intervention dose (g/day)						
≥3	9	−1.64 (−6.44, 3.15)	0.502	0.725	0.0%	0.673
<3	3	−3.37 (−9.80, 3.06)	0.304	0.449	0.0%	
Health status						
Healthy	7	−2.58 (−7.79, 2.62)	0.33	0.625	0.0%	0.856
Non-healthy	5	−1.87 (−7.58, 3.83)	0.52	0.615	0.0%	
Exercise						
Yes	6	−1.01 (−6.75, 4.73)	0.73	0.418	0.0%	0.565
No	6	−3.28 (−8.46, 1.89)	0.214	0.88	0.0%	
HMB supplementation on Triglycerides (mg/dL)						
Overall effect	11	−2.83 (−12.93, 7.26)	0.583	0.007	58.4%	
Baseline TG (mg/dL)						
<150	7	1.51 (−14.16, 17.19)	0.85	0.006	67.2%	0.19
>150	2	−28.25 (−69.91, 13.40)	0.184	0.124	57.8%	
Trial duration (week)						
≤8	7	−2.32 (−14.62, 9.98)	0.712	0.033	56.1%	0.794
>8	4	−5.85 (−29.34, 17.64)	0.625	0.022	68.8%	
Intervention type						
HMB	6	−0.26 (−20.65, 20.12)	0.98	0.018	63.3%	0.801
HMB-Ca	5	−3.37 (−16.38, 9.64)	0.611	0.036	61.0%	
Gender						
Both	2	11.40 (−0.94, 23.76)	0.07	0.763	0.0%	0.029
Male	7	−2.32 (−14.62, 9.98)	0.712	0.033	56.1%	
Female	2	−23.19 (−46.60, 0.20)	0.052	0.255	23.0%	
Intervention dose (g/day)						
≥3	8	1.59 (−11.38, 14.57)	0.809	0.010	61.9%	0.069
<3	2	−23.19 (−46.60, 0.20)	0.052	0.255	23.0%	
Health status						
Healthy	7	−2.32 (−14.62, 9.98)	0.712	0.033	56.1%	0.794
Non-healthy	4	−5.85 (−29.34, 17.64)	0.625	0.022	68.8%	
Exercise						
Yes	4	−12.86 (−43.70, 17.98)	0.414	0.159	42.0%	0.469
No	7	−0.77 (−11.58, 10.02)	0.888	0.007	66.3%	
HMB supplementation on LDL-C (mg/dL)						
Overall effect	12	0.12 (−3.02, 3.27)	0.937	0.375	7.1%	
Baseline LDL-C (mg/dL)						
<130	7	2.48 (−1.71, 6.67)	0.246	0.916	0.0%	0.201
>130	3	−5.62 (−17.33, 6.08)	0.346	0.052	66.2%	
Trial duration (week)						
≤8	7	−1.34 (−6.54, 3.86)	0.613	0.209	28.7%	0.372
>8	5	1.99 (−3.14, 7.13)	0.448	0.618	0.0%	
Intervention type						
HMB	7	0.06 (−6.93, 7.06)	0.986	0.183	32.1%	0.881
HMB-Ca	5	−0.54 (−4.46, 3.36)	0.783	0.592	0.0%	
Gender						
Both	3	6.56 (−1.70, 14.82)	0.12	0.997	0.0%	0.257
Male	7	−1.34 (−6.54, 3.86)	0.613	0.209	28.7%	
Female	2	−0.89 (−7.45, 5.67)	0.79	0.394	0.0%	
Intervention dose (g/day)						
≥3	8	0.68 (−2.66, 4.03)	0.69	0.824	0.0%	0.982
<3	3	0.60 (−5.26, 6.47)	0.84	0.422	0.0%	
Health status						
Healthy	7	−1.34 (−6.54, 3.86)	0.613	0.209	28.7%	0.372
Non-healthy	5	1.99 (−3.14, 7.13)	0.448	0.618	0.0%	
Exercise						
Yes	5	1.10 (−4.54, 6.74)	0.702	0.646	0.0%	0.682
No	7	−0.48 (−5.56, 4.59)	0.851	0.164	34.7%	
HMB supplementation on HDL-C (mg/dL)						
Overall effect	12	−0.78 (−2.04, 0.48)	0.225	0.413	3.2%	
Baseline HDL-C (mg/dL)						
<50	6	−1.42 (−3.74, 0.88)	0.227	0.204	30.9%	0.653
>50	4	−0.64 (−3.14, 1.84)	0.611	0.556	0.0%	
Trial duration (week)						
≤8	7	−0.90 (−2.86, 1.05)	0.366	0.159	35.3%	0.878
>8	5	−0.66 (−2.97, 1.64)	0.572	0.721	0.0%	
Intervention type						
HMB	7	−1.60 (−3.74, 0.53)	0.141	0.265	21.5%	0.227
HMB-Ca	5	0.07 (−1.61, 1.76)	0.933	0.786	0.0%	
Gender						
Both	3	−2.18 (−6.65, 2.28)	0.338	0.769	0.0%	0.73
Male	7	−0.90 (−2.86, 1.05)	0.366	0.159	35.3%	
Female	2	−0.11 (−2.81, 2.58)	0.935	0.33	0.0%	
Intervention dose (g/day)						
≥3	8	−0.09 (−1.66, 1.47)	0.905	0.637	0.0%	0.831
<3	3	−0.42 (−3.03, 2.18)	0.749	0.415	0.0%	
Health status						
Healthy	7	−0.90 (−2.86, 1.05)	0.366	0.159	35.3%	0.878
Non-healthy	5	−0.66 (−2.97, 1.64)	0.572	0.721	0.0%	
Exercise						
Yes	5	−2.20 (−4.50, 0.09)	0.06	0.855	0.0%	0.171
No	7	−0.16 (−1.96, 1.63)	0.857	0.243	24.4%	

WMD, weighted mean differences; CI, confidence interval; BMI, body mass index; LDL-C, low-density lipoprotein cholesterol; HDL-C, high-density lipoprotein cholesterol; HMB, β-hydroxy-β-methylbutyrate.

#### 3.4.2 Effect of HMB supplementation on TG

According to the 11 effect sizes compared to control groups, HMB supplementation did not significantly reduce TG levels (WMD: −2.83 mg/dL 95% CI: −12.93 to 7.27; *p* = 0.58; Figure 2B). Additionally, a high degree of heterogeneity was detected between the included trials (I^2^ = 58.4%, *p* = 0.01). Subgroup analysis of HMB supplementation on TG is presented in Table 3.

#### 3.4.3 Effect of HMB supplementation on LDL-C

The overall results from evaluating 12 effect sizes indicated that HMB supplementation had no significant effect on LDL-C levels compared to control groups (WMD: 0.13 mg/dL; 95%CI: −3.02 to 3.28; mg; *p* = 0.94; Figure 2C). Moreover, no significant heterogeneity was observed among the included studies (I^2^ = 7.1%, *p* = 0.37). Subgroup analysis of HMB supplementation on LDL-C is shown in Table 3.

#### 3.4.4 Effect of HMB supplementation on HDL-C

After evaluating 12 effect sizes, it was found that HMB supplementation did not change HDL-C levels significantly (WMD: −0.78 mg/dL; 95%CI: −2.04 to 0.48; *p* = 0.22; Figure 2D). In addition, there was no significant heterogeneity among the included trials (I^2^ = 3.2%, *p* = 0.41). Subgroup analysis of HMB supplementation on HDL-C are presented in Table 3.

### 3.5 Sensitivity analysis and publication bias

The impact of each study on the overall effect size was determined by excluding each study from the analysis, respectively. The overall effect size was not significantly altered when each article was omitted. Upon examination of the funnel plots and conducting Egger’s test, it was observed that there is a notable publication bias in studies evaluating the effect of HMB supplementation on TC levels (*p* = 0.01). However, among the studies examining other outcomes, no significant publication bias was detected (Supplementary Figure S3).

### 3.6 Certainty assessment

The GRADE analysis demonstrated that the quality of evidence about the effect of HMB supplementation on LDL-C and HDL-C levels was identified as moderate due to the presence of serious limitations in imprecision. Furthermore, the quality of evidence for TG and TC levels was downgraded to low due to the presence of serious limitations in inconsistency, imprecision, and publication bias (Table 4).

**Table 4:** GRADE profile of HMB supplementation on lipid profile.

Outcomes	Risk of bias	Inconsistency	Indirectness	Imprecision	Publication Bias	Quality of evidence
Total cholesterol	No serious limitations	No serious limitations	No serious limitations	Serious limitations ^2^	Serious limitations^3^	⊕ ⊕ ◯◯ Low
Triglycerides	No serious limitations	Serious limitations ^1^	No serious limitations	Serious limitations	No serious limitations	⊕ ⊕ ◯◯ Low
LDL-C	No serious limitations	No serious limitations	No serious limitations	Serious limitations	No serious limitations	⊕ ⊕ ⊕◯ Moderate
HDL-C	No serious limitations	No serious limitations	No serious limitations	Serious limitations	No serious limitations	⊕ ⊕ ⊕◯ Moderate

There is high heterogeneity (I^2^ > 40%). There is no significant effect of HMB supplementation. There is a significant publication bias.

## 4 Discussion

To the best of our knowledge, this is the first meta-analysis to investigate the effects of HMB supplementation on lipid profiles in adults. The results of our meta-analysis, conducted on 10 RCTs with a total of 421 (intervention group: 227, control group: 194) adult participants, revealed no beneficial effects of HMB supplementation on TC (WMD: −2.26 mg/dL; 95%CI: −6.11 to 1.58; *p* = 0.25), TG (WMD: −2.83 mg/dL 95% CI: −12.93 to 7.26; *p* = 0.58), LDL-C (WMD: 0.12 mg/dL; 95%CI: −3.02 to 3.27; mg; *p* = 0.94) and HDL-C (WMD: −0.78 mg/dL; 95%CI: −2.04 to 0.48; *p* = 0.22) in adults. Moreover, subgroup analysis revealed that HMB and HMB-Ca supplements had no effects on the lipid profile in all subjects.

In recent years, HMB has been used as one of the nutritional supplements used by athletes to adjust homeostasis and increase lipolysis and fat-free mass. Several studies have shown that supplementation with HMB alters cholesterol synthesis in the liver by converting it to HMG-CoA (9, 38–40). Different studies have been performed on the effects of HBM on weight loss, lipid profile, and muscle strength.

In a recent study, Ansari et al. (2021) investigated the effect of HMB supplementation on lipid profile and some indicators of physical fitness in beginner bodybuilders. According to their findings, compared with the placebo group, the HBM group demonstrated a significant reduction in triglyceride levels, but for other factors such as LDL, HDL, LDL/HDL, and cholesterol, there was no significant difference. An additional study conducted in 2001 by Coelho and Carvalho showed that supplementation with HMB significantly reduced LDL levels in hypercholesterolemic patients (12).

An analysis of nine studies published in 2000 by Nissen et al. examined data from subjects who received 3 grams of HMB per day for 3 to 8 weeks (including men and women, young and older, and athletes and non-athletes). According to the results, supplementation with HMB decreased TC and LDL cholesterol levels. However, HMB has no significant effect on LDL levels in people with normal cholesterol levels (14). This may be interpreted as HMB lowering LDL levels in the presence of high cholesterol levels. Conversely, some studies have found that HMB supplementation does not significantly affect blood cholesterol levels (41).

Arazi et al. studied HMB supplementation and cardiovascular risk factors (hematological parameters, blood pressure, and blood lipid levels) in 2015. Although their results showed the beneficial changes in blood lipids in both groups, no significant differences were found between the two groups concerning blood lipids (31). The results of another study conducted by Gallagher et al. in 2000 indicated that the use of varying doses of HMB (0, 3, or 6 g) did not result in significant changes to the lipid profile levels following 8 weeks of resistance training in untrained men (38). These findings were in line with our findings of the present study, as HMB supplementation had no significant effect on blood lipids. Differences in results between different studies may be due to differences in the type of experimental design, quantity and intensity of exercise, health status of the participants, duration of the experiment, supplemental formulas, methods of evaluating variables and statistical analysis methods.

The background diet of subjects indeed plays a crucial role in the overall effects of HMB supplementation, given that HMB is a metabolite of leucine. It is important to note that most of the included studies managed to control for dietary intake to some extent, often using standardized meal plans during the intervention period. For example, the study by Yang et al. and Espina et al. monitored and controlled participants’ diets to mitigate the confounding effects of dietary variations (30, 35). It was necessary to apply this control in order to reduce the confounding effects of dietary variations, particularly protein and leucine intake, which can have an impact on endogenous HMB concentrations (13). However, it is acknowledged that not all studies could perfectly control for dietary habits, and this represents a limitation of the review. Further, it is suggested that future studies should include more rigorous dietary controls more precisely to account for such variables (40).

One proposed mechanism of action is that HMB may inhibit the activity of enzymes involved in lipolysis, such as hormone-sensitive lipase and adipose tissue triglyceride lipase. This inhibition ultimately leads to reduced breakdown of triglycerides into free fatty acids, thereby reducing the release of triglycerides into the circulation (16). Additionally, HMB may promote fat oxidation by increasing the expression of genes involved in fatty acid metabolism. By stimulating the oxidation of free fatty acids, HMB may contribute to the reduction in triglyceride levels (42, 43). Furthermore, it could indirectly affect lipid metabolism through other pathways. HMB supplementation is known to stimulate muscle protein synthesis. Increased muscle mass is often associated with a higher metabolic rate, potentially leading to favorable changes in lipid profiles (40). Additionally, HMB might influence inflammatory markers, which are linked to cardiovascular health and cholesterol regulation (44). Future research needs to explore these potential indirect effects in greater detail (45).

### 4.1 Safety and optimal supplementation dosage

The supplementation of HMB has been extensively studied, with evidence suggesting that an optimal dosage of 3 grams per day maximizes strength and lean body mass gains, while higher doses, such as 6 grams per day, do not confer additional benefits (38, 40). Although there is a theoretical basis for skeletal muscles being unable to absorb higher concentrations of HMB, thus explaining the lack of further gains at higher doses (38), the safety of HMB at various dosages has been well-documented. Studies have shown that even at doses up to 50 grams per day in rat models, normalized to human equivalent dosing, no adverse effects were observed, and human studies have corroborated these findings, reporting no negative impacts on cholesterol, blood glucose, liver, or kidney functions at dosages of up to 6 grams per day for extended periods (46). Consequently, HMB is considered a safe supplement within the studied dosage range, potentially enhancing health markers without adverse effects (38, 47).

### 4.2 Strengths and limitations

This systematic review and meta-analysis has several strengths which include an acceptable number of studies. In addition, the majority of the studies we included were considered to be of high quality according to the Cochrane risk of bias tool. As the studies were conducted in different regions across the world, our findings can be applied to adult populations globally; however, this also highlights the need for future research to explore the impact of ethnicity on HMB supplementation outcomes, since the baseline HMB concentration might differ among various ethnic groups. Furthermore, the search was not limited to a particular time. There are some limitations to this study that need to be acknowledged. One of the primary limitations is the potential heterogeneity among studies included in the systematic reviews and meta-analyses. Different study designs, populations, and intervention protocols may have influenced the observed results, making it difficult to draw definitive conclusions. Additionally, confounding factors, such as exercise and diet, can potentially influence lipid profile changes observed with HMB supplementation. These factors cannot be adequately controlled for all studies included in the systematic reviews and meta-analyses, making it difficult to attribute the observed effects solely to HMB. To ensure the quality and comparability of the included studies, we restricted our analysis to English-language articles. This language limitation may have resulted in the exclusion of relevant studies published in other languages, potentially limiting the generalizability of our findings. In addition, some of the included studies did not clearly report the details of their randomization or blinding of the outcome assessment process. Lastly, most outcomes were moderate to low, according to the GRADE.

## 5 Conclusion

In conclusion, this systematic review and meta-analysis suggested that HMB supplementation does not significantly alter lipid profiles, including TC, TG, LDL-C, and HDL-C levels. Further research involving larger, more rigorously conducted studies with long-term intervention periods is necessary in order to fully investigate the mechanisms of action and potential clinical significance of HMB.

## Data Availability

The original contributions presented in the study are included in the article/Supplementary material, further inquiries can be directed to the corresponding author.

## Footnotes

^1^https://www.gradeworkinggroup.org/
