# Supplementary material for: Effects of β-hydroxy-β-methylbutyrate (HMB) supplementation on lipid profile in adults: a GRADE-assessed systematic review and meta-analysis of randomized controlled trials
Source: Front Nutr. 2024 Sep 25;11:1451282. doi: 10.3389/fnut.2024.1451282 (PMC11462179; doi:10.3389/fnut.2024.1451282)
Supplement: Supplementary file 2 [file Data_Sheet_1.docx]

**Title page:**

**Effects of β-Hydroxy-β-Methylbutyrate (HMB) supplementation on lipid profile in adults: A GRADE-assessed systematic review and meta-analysis of randomized controlled trials**

Behrad Sadeghi ^1^, Hossein Bahari ^2^, Hannane Jozi ^3^, Mohammad Ali-Hasanzadeh ^4^, Dorna Hashemi ^5^, Mohammad Vesal Bideshki ^3,6*^

^1^ Department of Agricultural, Forest and Food Science (DISAFA), University of Torino, Grugliasco, Torino 10095, Italy.

^2^ Transplant Research Center, Clinical Research Institute, Mashhad University of Medical Sciences, Mashhad, Iran.

^3^ Student Research Committee, Tabriz University of Medical Sciences, Tabriz, Iran.

^4^ Department of Immunology, School of Medicine, Jiroft University of Medical Sciences, Jiroft, Iran.

^5^ Department of Food Science and Technology, Sarvestan Branch, Islamic Azad University, Sarvestan, Iran.

^6^ Department of Biochemistry and Diet Therapy, School of Nutrition and Food Science, Tabriz University of Medical Sciences, Tabriz, Iran.

***Corresponding author:**

**Mohammad Vesal Bideshki**

Nutritional Sciences

Tabriz University of Medical Sciences,

Address: Attar-Neishaburi St., Golgasht Alley, Azadi Blvd., Tabriz, Iran.

Fax Number: +984133340634, Phone Number: +989135373792

Email: [Bideshkim@tbzmed.ac.ir](mailto:Bideshkim@tbzmed.ac.ir)

**(A)**

**(B)**

**(C)**

**(D)**

Figure s1. A-D. Assessment of publication bias in the impact of β-Hydroxy-β-Methylbutyrate supplementation on lipid profile in adults.


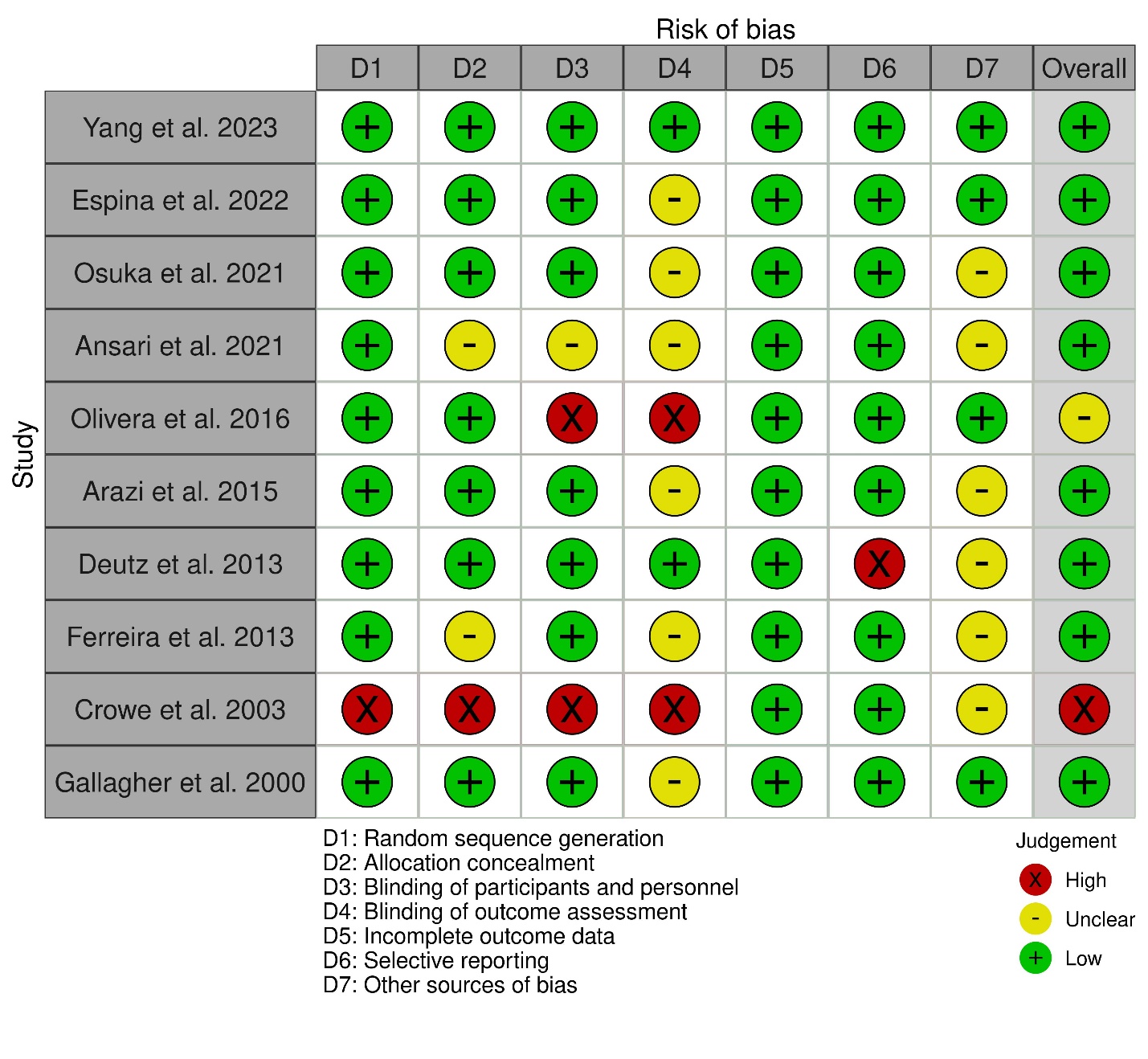

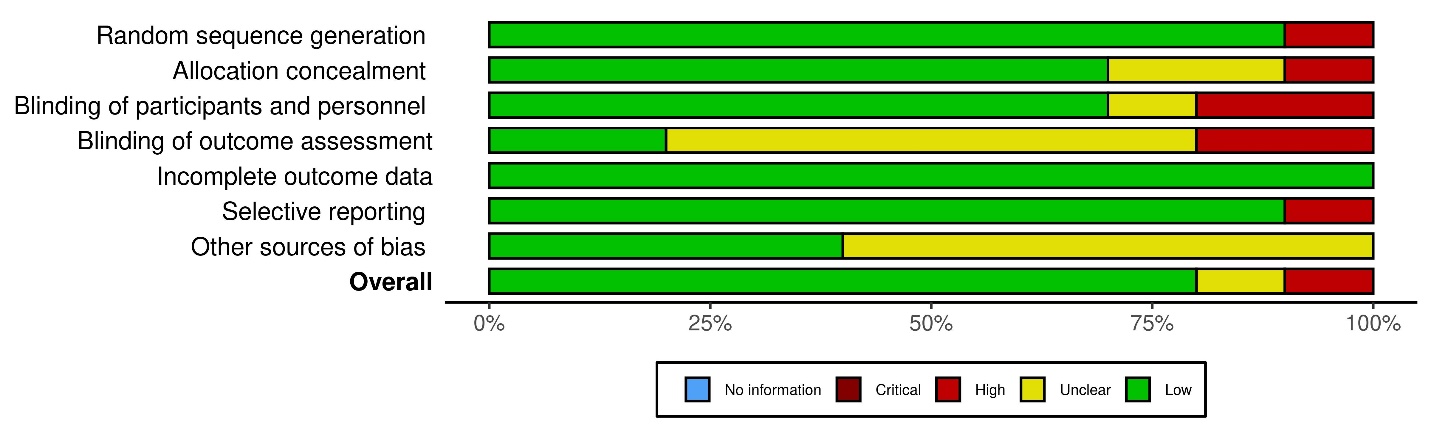


Figure s2. *Traffic light plot summarizing the risk of bias for each outcome assessed using the Cochrane Risk of Bias 2 (ROB2) tool. The five domains evaluated are: randomization, deviations from intended interventions, missing outcome data, measurement of outcomes, and selection of the reported result. Green indicates low risk, yellow indicates some concerns, and red indicates high risk of bias.*

**Table s1. Search strategy in databases**

| **Database** | **Strategy** | **Number** |
| --- | --- | --- |
| **PubMed** | (("beta-hydroxy beta-methylbutyrate") OR ("beta Hydroxy beta methylbutyric acid") OR ("hydroxy methylbutyrate") OR ("3-hydroxyisovaleric acid") OR ("b-hydroxybutyric acid") OR ("HMB") OR ("beta methylbutyrate") OR ("b-hydroxyb-methylbutyrate") OR ("sport supplements") OR ("hydroxy methylbutyrate") OR ("leucine metabolite") OR ("hmb fa") OR ("hmb ca") OR ("beta-hydroxy-beta-methylbutyrate free acid") OR ("beta-hydroxyisovaleric acid"[Supplementary Concept])) AND (("lipid profile") OR ("Total Cholesterol") OR ("HIGH DENSITY LIPOPROTEIN") OR ("Low-Density Lipoprotein") OR ("serum lipid") OR ("plasma lipid") OR ("plasma fatty acid") OR ("very low density lipoproteins") OR ("Apolipoproteins") OR ("Very-low density lipoprotein") OR ("Chylomicrons") OR ("triglyceride") OR ("Cholesterol") OR ("dyslipidemia") OR ("Cholesterol") OR "lipoproteins, hdl"[MeSH Terms] OR "cholesterol, ldl"[MeSH Terms] OR "Triglycerides"[MeSH Terms] OR "Apolipoproteins B"[MeSH Terms] OR "Fatty Acid-Binding Proteins"[MeSH Major Topic] OR "Fatty Acid-Binding Proteins"[MeSH Terms] OR "cholesterol, vldl"[MeSH Terms] OR "lipoproteins, vldl"[MeSH Terms] OR "lipoproteins, idl"[MeSH Terms] OR "Apolipoproteins"[MeSH Terms] OR "Apolipoproteins A"[MeSH Terms] OR "Chylomicrons"[MeSH Terms] OR "Lipids"[MeSH Terms] OR "Dyslipidemias"[MeSH Terms]) AND ("Randomized Controlled Trial" OR "Clinical Trial "OR "RCT" OR "Randomized Clinical Trial "OR Randomized OR Randomly OR Clinical OR Intervention OR Placebo OR Trial OR "Double blinded "OR "Double Blind Method" OR blind OR Control OR Random OR assignment OR "single blinded "OR "single-blind method" OR "Double-blind method "OR parallel OR "cross-over study") NOT ("in-vitro" OR "in-vivo" OR rabbit OR mouse OR rat OR mice OR cell OR monkey OR pig OR cow OR chicken OR goat OR sheep OR duck OR bacteria OR cat OR shrimp OR rooster OR warthogs OR broiler OR crab OR fowl) | **127** |
| **Scopus** | ( TITLE-ABS-KEY ( "beta-hydroxy beta-methylbutyrate" ) OR TITLE-ABS-KEY ( "&#946;-hydroxy &#946;-methylbutyrate" ) OR TITLE-ABS-KEY ( "beta Hydroxy beta methylbutyric acid" ) OR TITLE-ABS-KEY ( "hydroxy methylbutyrate" ) OR TITLE-ABS-KEY ( "3-hydroxyisovaleric acid" ) OR TITLE-ABS-KEY ( "b-hydroxybutyric acid" ) OR TITLE-ABS-KEY ( "HMB" ) OR TITLE-ABS-KEY ( "beta methylbutyrate" ) OR TITLE-ABS-KEY ( "b-hydroxyb-methylbutyrate" ) OR TITLE-ABS-KEY ( "sport supplements" ) OR TITLE-ABS-KEY ( "hydroxy methylbutyrate" ) OR TITLE-ABS-KEY ( "leucine metabolite" ) OR TITLE-ABS-KEY ( "HMB-FA" ) OR TITLE-ABS-KEY ( "HMB FA" ) OR TITLE-ABS-KEY ( "HMB-CA" ) OR TITLE-ABS-KEY ( "HMB CA" ) OR TITLE-ABS-KEY ( "beta-hydroxy-beta-methylbutyrate free acid" ) ) AND ( TITLE-ABS-KEY ( "lipid profile" ) OR TITLE-ABS-KEY ( "Total Cholesterol" ) OR TITLE-ABS-KEY ( "TC" ) OR TITLE-ABS-KEY ( "HIGH DENSITY LIPOPROTEIN" ) OR TITLE-ABS-KEY ( "HDL" ) OR TITLE-ABS-KEY ( "Low-Density Lipoprotein" ) OR TITLE-ABS-KEY ( "LDL" ) OR TITLE-ABS-KEY ( "TG" ) OR TITLE-ABS-KEY ( "apo B" ) OR TITLE-ABS-KEY ( "serum lipid" ) OR TITLE-ABS-KEY ( "plasma lipid" ) OR TITLE-ABS-KEY ( "plasma fatty acid" ) OR TITLE-ABS-KEY ( "very low density lipoproteins" ) OR TITLE-ABS-KEY ( "apolipoproteins" ) OR TITLE-ABS-KEY ( "Apo A" ) OR TITLE-ABS-KEY ( "VLDL" ) OR TITLE-ABS-KEY ( "Very-low density lipoprotein" ) OR TITLE-ABS-KEY ( "chylomicrons" ) OR TITLE-ABS-KEY ( "triglyceride" ) OR TITLE-ABS-KEY ( "lipids" ) OR TITLE-ABS-KEY ( "cholesterol" ) OR TITLE-ABS-KEY ( "dyslipidemia" ) ) AND ( TITLE-ABS-KEY ( "Randomized Controlled Trial" ) OR TITLE-ABS-KEY ( "Clinical Trial" ) OR TITLE-ABS-KEY ( "RCT" ) OR TITLE-ABS-KEY ( "Randomized Clinical Trial" ) OR TITLE-ABS-KEY ( randomized ) OR TITLE-ABS-KEY ( randomly ) OR TITLE-ABS-KEY ( clinical ) OR TITLE-ABS-KEY ( intervention ) OR TITLE-ABS-KEY ( placebo ) OR TITLE-ABS-KEY ( trial ) OR TITLE-ABS-KEY ( "Double blinded" ) OR TITLE-ABS-KEY ( "Double Blind Method" ) OR TITLE-ABS-KEY ( blind ) OR TITLE-ABS-KEY ( control ) OR TITLE-ABS-KEY ( random ) OR TITLE-ABS-KEY ( assignment ) OR TITLE-ABS-KEY ( "single blinded" ) OR TITLE-ABS-KEY ( "single-blind method" ) OR TITLE-ABS-KEY ( "Double-blind method" ) OR TITLE-ABS-KEY ( parallel ) OR TITLE-ABS-KEY ( "cross-over study" ) AND NOT TITLE-ABS-KEY ( "in-vitro" ) OR TITLE-ABS-KEY ( "in-vivo" ) OR TITLE-ABS-KEY ( rabbit ) OR TITLE-ABS-KEY ( mouse ) OR TITLE-ABS-KEY ( rat ) OR TITLE-ABS-KEY ( mice ) OR TITLE-ABS-KEY ( cell ) OR TITLE-ABS-KEY ( monkey ) OR TITLE-ABS-KEY ( pig ) OR TITLE-ABS-KEY ( cow ) OR TITLE-ABS-KEY ( chicken ) OR TITLE-ABS-KEY ( goat ) OR TITLE-ABS-KEY ( sheep ) OR TITLE-ABS-KEY ( duck ) OR TITLE-ABS-KEY ( bacteria ) OR TITLE-ABS-KEY ( cat ) OR TITLE-ABS-KEY ( shrimp ) OR TITLE-ABS-KEY ( rooster ) OR TITLE-ABS-KEY ( warthogs ) OR TITLE-ABS-KEY ( broiler ) OR TITLE-ABS-KEY ( crab ) OR TITLE-ABS-KEY ( fowl ) ) | **268** |
| **Web of Science** | (((ALL=("beta-hydroxy beta-methylbutyrate" OR "β-hydroxy β-methylbutyrate" OR "beta Hydroxy beta methylbutyric acid" OR "hydroxy methylbutyrate" OR "3-hydroxyisovaleric acid" OR "b-hydroxybutyric acid" OR "HMB" OR "beta methylbutyrate" OR "b-hydroxyb-methylbutyrate" OR "sport supplements" OR "hydroxy methylbutyrate" OR "hydroxy methylbutyrate" OR "leucine metabolite" OR "HMB-FA" OR "HMB FA" OR "HMB-CA" OR "HMB CA" OR "beta-hydroxy-beta-methylbutyrate free acid")) AND ALL=("lipid profile" OR "Total Cholesterol" OR "TC" OR "HIGH DENSITY LIPOPROTEIN" OR "HDL" OR "Low-Density Lipoprotein" OR "LDL" OR "TG" OR "apo B" OR "serum lipid" OR "plasma lipid" OR "plasma fatty acid" OR "very low density lipoproteins" OR "apolipoproteins" OR "Apo A" OR "VLDL" OR "Very-low density lipoprotein" OR "chylomicrons " OR "lipids " OR "triglyceride" OR "cholesterol" OR "dyslipidemia")) AND ALL=("Randomized Controlled Trial" OR "Clinical Trial" OR "RCT" OR "Randomized Clinical Trial" OR "Randomized" OR "Randomly" OR "Clinical" OR "Intervention" OR "Placebo" OR "Trial" OR "Double blinded" OR "Double Blind Method" OR "blind" OR "Control" OR "Random" OR "assignment" OR "single blinded" OR "single-blind method" OR "Double-blind method" OR "parallel" OR "cross-over study")) NOT ALL=("in-vitro" OR "in-vivo" OR "rabbit" OR "mouse" OR "chicken" OR "duck" OR "mice" OR "rat" OR "monkey" OR "pig" OR "cell" OR "cow" OR "goat" OR "bacteria" OR "sheep" OR "cat" OR "shrimp" OR "rooster" OR "warthogs" OR "broiler" OR "crab" OR "fowl") | **164** |

**Duplicate: 68 items**
